# Case report and literature review: Hemophagocytic lymphohistiocytosis in a pregnant woman with systemic lupus erythematosus with *Syntaxin 11* gene defect

**DOI:** 10.3389/fonc.2022.937494

**Published:** 2022-07-28

**Authors:** Wei Ren, Siyuan Yang, Haiying Liu, Zhenglun Pan, Zhao Li, Peng Qiao, Hui Ma

**Affiliations:** ^1^ Department of Gynaecology and Obstetrics, Qilu Hospital, Cheeloo College of Medicine, Shandong University, Qingdao, China; ^2^ College of Clinical Medicine, Weifang Medical University, Weifang, China; ^3^ Department of Rheumatism and Immunology, Qilu Hospital, Cheeloo College of Medicine, Shandong University, Qingdao, China; ^4^ Department of Hematology, Qilu Hospital, Cheeloo College of Medicine, Shandong University, Qingdao, China

**Keywords:** hemophagocytic lymphohistiocytosis, systemic lupus erythematosus, pregnant, *Syntaxin 11*, cyclosporine A

## Abstract

Hemophagocytic lymphohistiocytosis is an extremely rare occurrence during pregnancy. Early recognition of its signs and symptoms is critical for early intervention, and delays in diagnosis may be life-threatening. A 23-year-old nulliparous woman presented with a persistent fever as high as 39°C with bilateral edema of the lower limbs at 24 weeks of gestation. Typical laboratory findings included pancytopenia, high triglycerides, ferritin, transaminases, bilirubin, and hypoproteinemia. Active systemic lupus erythematosus was diagnosed using an autoimmune work-up and a Systemic Lupus Erythematosus Disease Activity Index 2000 score of 17 points. Her bone marrow aspirate revealed prominent hemophagocytosis; hence, HLH was confirmed. Genetic tests showed mutations in *Syntaxin 11* mutations. Considering the potential impact of drugs on the fetus, the patient and her family members chose to terminate the pregnancy through medical induction of labor. Afterwards, her condition improved with immunosuppressive therapy.

## Introduction

Hemophagocytic lymphohistiocytosis (HLH), as a rare and severe clinical syndrome, is characterized by excessive immune cell activation with significant hemophagocytic activity in the bone marrow that may cause a cytokine storm (1–3). In 1952, the case was first published (4). Primary HLH or familial HLH (FHLH), which is a genetically heterogeneous disorder, typically manifests during infancy and childhood with a rapidly fatal outcome if left untreated (5). It is an autosomal recessive condition in which the defective gene causes a reduction in NK cell and cytotoxic T lymphocyte function, leading to excessive immune activation. Approximately 90% of all patients have the onset of the disease before 2 years of age (6, 7). Secondary HLH, also known as macrophage activation syndrome (MAS), shares the same clinical and biological characteristics as FHLH but lacks a family history of disease or genetic defects, which contributes to a high risk of developing HLH (8). It could be caused by several diseases, including infection, immunodeficiency syndromes, hematological malignancies, rheumatologic illnesses, and autoimmune diseases (9). Cardinal signs and symptoms of HLH include prolonged high fever, cytopenia, hepatosplenomegaly, liver dysfunction, elevated ferritin, triglycerides, serum transaminases, and bilirubin (10, 11). We report a rare case of reactive HLH in a pregnant woman with acute systemic lupus erythematosus (SLE) and a *Syntaxin 11 (STX11)* gene defect. A review of the literature was further performed using PubMed and the CNKI database to clarify its pathogenesis, clinical manifestation, diagnosis, and treatment.

## Case Presentation

A 23-year-old woman (gravida 1, para 0) weighing 60 kg was transferred to our hospital due to persistent fever up to 39°C for three days with bilateral edema of the lower limbs at 24 weeks of gestation. She had no history of autoimmune disease, and her family history was unremarkable. Upon examination, the following vitals were obtained: a temperature of 38.1 °C (febrile), a resting heart rate of 105 beats/min (tachycardic), and a blood pressure of 97/58 mmHg (hypotensive). She received fluid resuscitation and empiric antibiotics. The dermatological and neurological examinations were unremarkable. A physical examination showed marked hepatosplenomegaly; however, no arthritis was noted. Pertinent laboratory findings indicated pancytopenia including the following: hemoglobin (HGB) of 9.3 g/dl; platelet (PLT) count of 80 × 10^9^/L; and leukocyte (WBC) count of 3.45 × 10^9^/L; slight liver dysfunction and elevated lactate dehydrogenase (LDH) level of 477 U/L (normal range [NR], 91–245); C-reactive protein (CRP) level of 17.26 mg/dl; and decreased albumin (ALB) level of 26 g/L (NR, 35–55). The following findings were also obtained: positive antinuclear antibody (1:1,000), anti-SSA >200 AU/ml (NR, 0–20), antidouble-stranded DNA antibody of 34.5 IU/ml (NR, 0–20), and negative anticardiolipin antibodies; complement C3 level of 0.32 g/L (NR, 0.9–1.8), C4 level of 0.1 g/L (NR, 0.1–0.4); EB virus capsid antigen IgA/IgM/IgG antibody, early antigen IgM, and nuclear antigen IgG negative. Serological investigations showed no evidence of active Epstein–Barr virus (EBV), cytomegalovirus disease (CMV), herpes simplex, leptospira, tuberculosis, hepatitis B, hepatitis C, VDRL, and human immunodeficiency virus (HIV); immunoglobulin IgE markedly elevated to 588 IU/ml (NR, 0–100); and urine occult blood and proteinuria. Other active infections were excluded. Abdominal ultrasound demonstrated a splenic swelling of 5.8 cm thick. Transthoracic echocardiography showed no obvious abnormalities. A diagnostic procedure for active SLE was purchased. The patient fulfilled the 2019 American College of Rheumatology (ACR) classification criteria: 24 points for SLE with a high activity index (SLEDAI of 15). As the patient was listless, we performed relative tests to rule out lupus encephalopathy. Neurological examinations, cerebrospinal fluid tests, magnetic resonance imaging, and electroencephalography were normal. The patient also showed no symptoms of lupus encephalopathy. Treatments including broad-spectrum antibiotics, intravenous immunoglobulin (15 g/day) with maintenance of high-dose prednisolone (40 mg/day), hydroxychloroquine 200 mg twice a day, and aspirin 50 mg once a day were started. However, the condition of the patient did not improve. Three days after admission, she developed butterfly erythema on her face and palm, and intermittent hyperpyrexia. Furthermore, the leukocyte count and hemoglobin decreased progressively; serum levels of alanine aminotransferase and aspartate aminotransferase were elevated to 59 U/L and 127 U/L, respectively; and serum ferritin, 1,859.80 ng/ml (NR, 11.0–306.8), NT-pro BNP, PCT, and D-Dimer were also elevated (**Table 1**). The 24-hour urine protein was 2.737 g/24 h (NR, 0–0.15). The SLEDAI score was estimated to be 17. The multidisciplinary consultation is recommended to replace prednisone with methylprednisolone pulse therapy (40 mg/day) in addition to 80 mg/day in the next 2 days. Moreover, the antibiotics were upgraded to meropenem. Although optimal management was provided, there was no relief of symptoms.

**Table 1:** Evolution of partial laboratory results.

Index	Unit	Day 1	Day 2	Day 4	Day 5	Day 6	Day 7	Day 8	Day 9	Day 10	Day 11	Day 12	Day 13	Day 14	Day 15	Day 16
**PCT**	ng/ml	–	0.91	4.3	3.11	2.25		0.68	0.58	–	–	0.34	0.44	–	0.12	0.11
**CRP**	mg/L	17.26	10.04	15.2	12.78	21.85	30.9	23.02	23.19	34.06	37.02	23.21	–	16.07	–	–
**Ferritin**	ng/ml	–	–	1859.8	–	–	–	–	–	955.9	–	828.4	791.8	941.82	–	–
**NT-proBNP**	Pg/ml	–	–	–	1,703	3225	3,702	3,941	3,464	–	–	–	5377	–	350	127
**ALB**	g/L	26	23.5	27.8	23.1	25.5	25.1	21.8	24.9	26.6	28.8	29.7	–	–	–	–
**LDH**	U/L	–	477	736	722	629	551	466	–	534	550	484	401	–	–	–
**D-Dimer**	ug/ml	7.63	9.55	12.77	20.54	–	14.46	–	–	10.08	11.51	19.64	23.45	–	–	–
**ALT** **AST**	U/L U/L	29 57	26 50	59 127	67 142	67 112	53 73	38 46	32 43	32 49	27 44	29 42	29 39	–	–	16 11
**TF**	g/L	–	–	2.04	–	–	–	–	–	–	–	–	–	2.31	–	–
NK cell activity	%	–	–	–	–	–	–	–	–	–	–	–	1.00	–	–	5.04
ESR	mm/h	54	54	–	–	–	111	–	–	–	–	16	–	–	–	–
proteinuria	g/L	3+	–	–	–	–	–	–	3+	–	3+	–	–	–	–	+
hematuria	–	3+	–	–	–	–	–	–	3+	–	3+	–	–	–	–	3+

**Figure 1:**
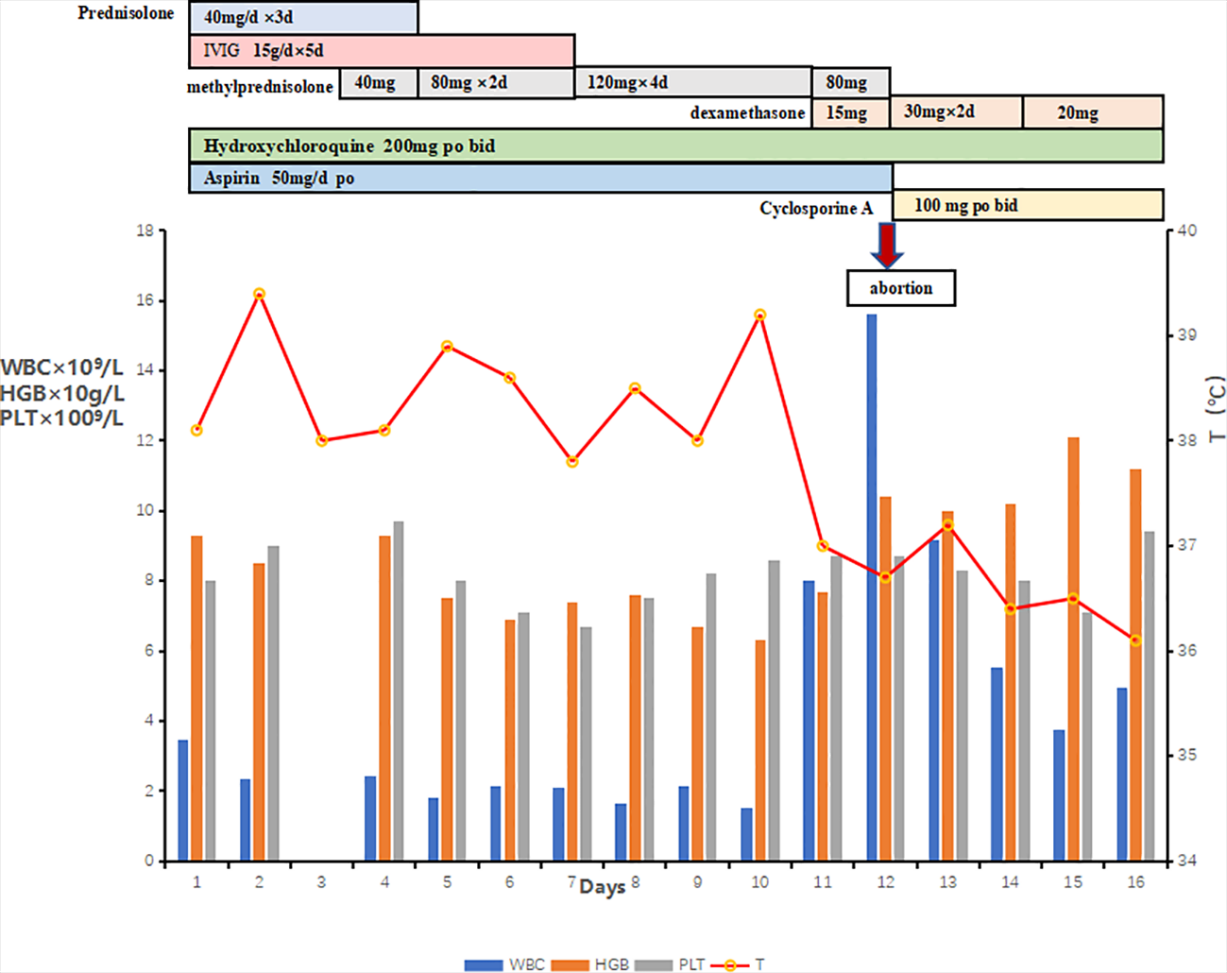
Medications administered, and changes in WBC, HGB, PLT, and body temperature are shown.

**Figure 2:**
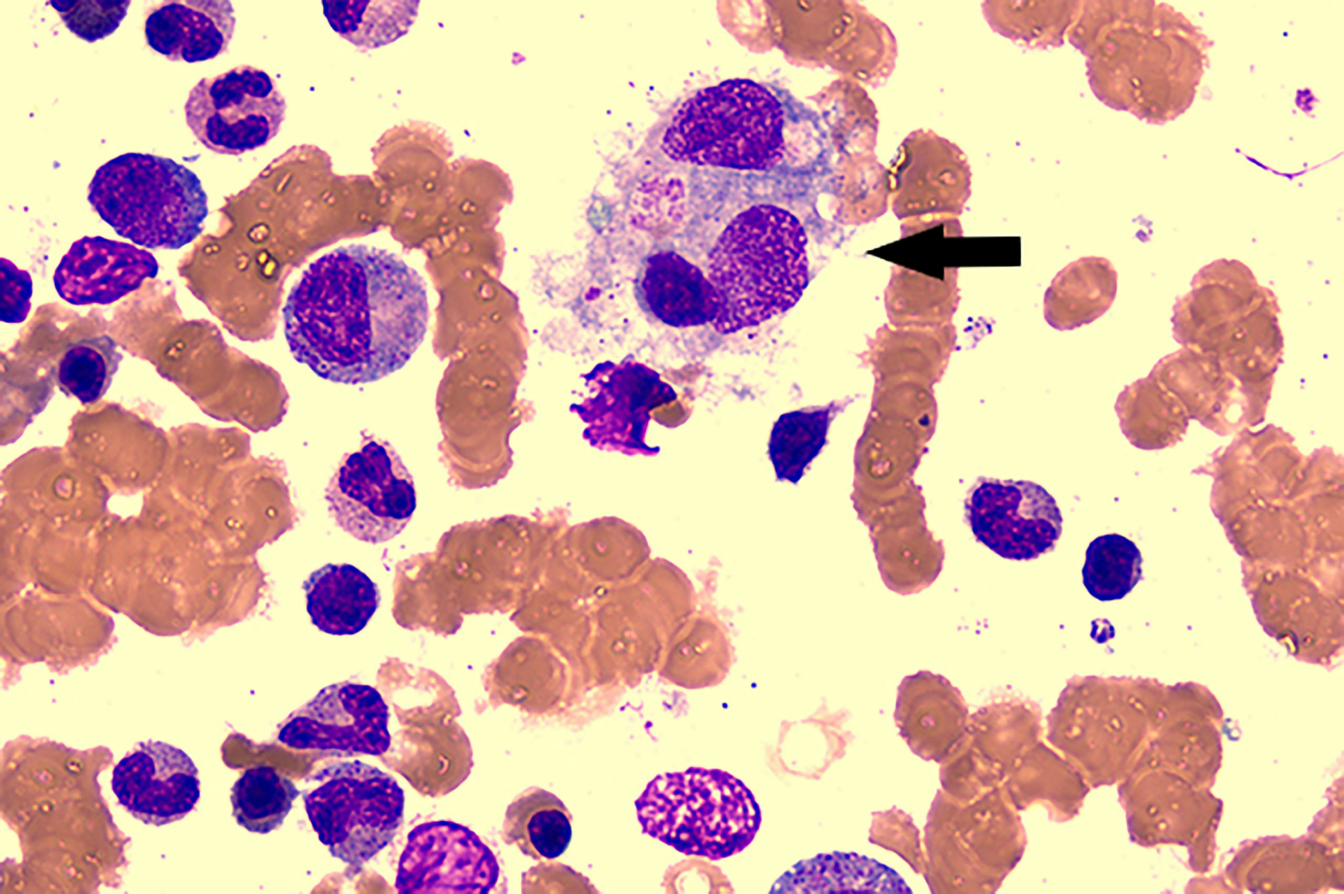
Bone marrow aspirate smear showed the evidence of hemophagocytosis; original magnification ×400(arrow).

On the sixth hospital day, the patient still suffered from daily fever (**Figure 1**) with further pancytopenia and elevated D-Dimer. The ALB decreased to 25.5 g/L. The dosage of methylprednisolone was adjusted to 80 mg and 40 mg in the morning and evening, respectively, according to the conditions for three consecutive days. Enoxaparin was administered to prevent thrombosis. She was transfused with red blood cells for anemia and was given albumin intravenously along with other symptomatic support treatments. A bone marrow biopsy revealed hemophagocytic cells (**Figure 2**). From these findings, a tentative diagnosis of SLE presenting as reactive HLH was made. Therefore, we organized the second multidisciplinary consultation. According to the laboratory findings, immunosuppressive therapy with dexamethasone at 15 mg/day (since methylprednisolone had been given 80 mg in the morning) and 30 mg/day on the first day and next 2 days, respectively, was found to be effective. The response to steroids was not immediately stimulated; hence, etoposide was considered. Since she was classified under US FDA pregnancy category D, the family of the patient refused its accompanied treatment options. Cyclosporine A is classified under class C, and informed consent was obtained to initiate 100 mg twice a day through oral administration. Afterwards, the fever, blood cell count, liver function, and coagulation dysfunction subsided gradually.

Owing to the use of multiple drugs during pregnancy and the unpredictable prognosis of the fetus, the patient decided to terminate her pregnancy and gave up rescuing the newborn. A drug induced labor operation was successfully completed under close monitoring. On the day of labor induction, the patient developed high blood pressure, and oral labetalol and nifedipine failed to control it. Therefore, intravenous pumping of Urapidil hydrochloride injection was performed to control hypertension. Enoxaparin was administered to prevent thrombosis at 12 h postpartum. Unfortunately, chest discomfort and suffocation appeared in the first day after abortion. Transthoracic echocardiography showed tricuspid regurgitation (mild-moderate), pulmonary valve regurgitation (mild-moderate), pulmonary hypertension (mild), pericardial effusion (mild), and pleural effusion (mild). A third multidisciplinary consultation was established to develop a better plan. Since pulmonary embolism was considered, the experts suggested that corticosteroids, cyclosporine, hydroxychloroquine, and enoxaparin should be continued on the basis of the original therapeutic schedule. Furthermore, the following laboratory tests improved soluble CD25 by 4,027.91 pg/ml (NR, 458–1,997) and low natural killer (NK) cell activity by 1.00 (NR, 8.1%–25.6%). Genetic tests described the *STX11* mutations. It was found to be positive for an exon 2, c.646C >A (p.R216s) heterozygous mutation. The placenta showed no microscopic abnormalities. Fortunately, the condition of the patient did not deteriorate further. The SLEDAI score was decreased to 10, including proteinuria, hematuria, and low complement protein3. Under our close supervision and careful treatment, the patient was discharged 2 weeks after the termination of the pregnancy. She chose to go to the local hospital for re-examination and continued to take prednisone 10 mg/day and cyclosporine 50 mg/day orally for 8 weeks, following the advice of the doctor. She has remained in remission for 7 months until now.

## Discussion

HLH is a disease with a high mortality rate since it is often misdiagnosed because of its complex clinical presentation. The annual incidence worldwide is estimated at one per 800,000, in which the majority of adult cases are Asian (12). Since no guidelines or expert consensus were achieved, it is useful to summarize all the relevant studies to provide a clearer strategy of the diagnosis, management, and therapy to clinicians. Here, we systematically searched all the related reports from PubMed and CNKI for HLH in pregnancy. The exclusion criteria included publications unrelated to the diagnosis of HLH-related pregnancy and not written in English. Sixty-six cases were included in the article, ranging from 1999 to 2022. Among these, 48 cases demonstrated a clear etiology. Sixty-five cases of secondary HLH found that infection (24/65) remained the primary factor, accounting for 37% of all pathogenic factors (*Staphylococcus epidermidis* 1, viscera leishmaniasis 1, Parvovirus B19 2, pericoronitis abscess 1, Tuberculosis 1, HSV-2 3, HIV 2, CMV 3, EBV 9, and HBV 1). EBV is the most common causative agent. Other causes included hematological malignancy (3/65), autoimmune diseases (13/65, SLE7), genetic factors (1/66), and 28% (n = 18) had no obvious underlying cause. Other infrequent causes include pregnancy-related conditions, pre-eclampsia, HELLP syndrome, and autoimmune hemolytic anemia. Only one case has been reported of genetic HLH. Wang et al. (13) reported that a 40-year-old woman was triggered twice during the first trimester of her first and second pregnancies with a novel exon 19, c.1607G >T (p.Arg536Leu) heterozygous mutation of the UNC13D gene. Familial HLH (FHLH) was linked to a number of defects in genes: Perforin (PRF1), Munc 13-4 (UNC13D), STX11, and Munc *18-2 (STXBP2*) (9). Based on the genetic etiology, FHLH has been subcategorized into 5 subtypes (FHLH-1 to FHLH-5) (14). FHLH type 1 has been credited to mutations in a gene located on chromosome 9 that is yet unidentified (15). FHLH type 2 was the first genetic defect discovered to be related to FHLH, which is due to homozygous mutations in the gene PRF1, which encodes a pore-forming protein called Perforin 1, that is vital for target cell lysis (16). Depending on some cohort studies, PRF1 mutations account for 20%–50% of FHL, with some variation between populations of different ethnic backgrounds (17). Mutations in the genes UNC13D, STX11, and STXBP2 account for FHLH types 3, 4, and 5, respectively (14). A third locus, 17q25, associated with FHLH type 3, thought to be attributed to the initiation of secretory particles and their fusion on plasma membranes, was first reported in 2003 (18). Over 50 types of mutations of UNC13D have been reported until now (17). The region on chromosome 6q24 maps to the STX11 gene encoding syntaxin 11 protein, expressed most prominently in phagocytes and antigen-presenting cells (19, 20). In 2009, a novel gene located on chromosome 19p was reported to be associated with FHLH. Based on this finding, zur Stadt et al. identified mutations in STXBP2 encoding for Munc18-2 (21). 14 different mutations of STXBP2 have been described. Similar to those with FHLH type 4, they appear to have a later onset compared with FHLH types 2 and 3 (22). In this study, the patient whose genetic tests described the *STX11* mutations had no history of childhood illness. She probably had a genetic predisposition that lowers the threshold for developing the disease. Some scholars argue that the etiology of pregnancy-induced HLH may be related to an immature placenta that releases trophoblast debris and soluble RNA or DNA of fetal origins into the maternal circulation. This might cause various immune disorders leading to cytokine storms, which are exacerbated by the deterioration of the clinical condition of the patient (23, 24). Subsequently, hemophagocytosis will lead to the failure of multiple organs, including the liver, brain, and bone marrow, which can be potentially fatal (25). We believe that the heterozygous STX11 mutation and SLE may predispose her to immune overactivation, and autoimmune diseases can worsen during pregnancy, which results in the clinical manifestations of adult-onset FHLH. A study reported that 0.9%–2.4% of patients with SLE develop MAS (26). While FHL and MAS were previously considered separate entities, in our case, they presented a continuum disease in which the genetic predisposition of the patient likely lowers the threshold for developing the disease. SLE and pregnancy likely acted as predisposing factors and triggers, respectively. Distinguishing MAS from SLE has been challenging as they share the same clinical and laboratory characteristics. The pathogenesis of MAS is thought to be the excessive stimulation of macrophages and cytotoxic T lymphocytes (CD8+ T cells) secreting a large number of cytokines, leading to cytokine storms (27). Unfortunately, few studies have addressed the individual diseases that cause HLH, the risk factors that lead to it, and the outcomes associated with it. The most identified trigger factor that led to the development of MAS in some studies was a lupus flare, which signifies that the flare itself is an independent risk factor for developing MAS. This is followed by infections in patients with SLE (28, 29). In Cohen et al.’s (30) study, increasing SLEDAI scores were found to be a risk factor for MAS development in SLE patients. Other studies showed that lupus flare itself, as well as time to onset and high SLEDAI scores, were crucial risk factors that led to the development of MAS (31).

From previous cases, only seven cases of pregnancy-related HLH secondary to SLE (**Table 2**) were reported, with a median age of 27 years (range 20–31). Three cases (43%) were in the early trimester and middle trimester and one case (14%) in the late trimester. Corticosteroid therapy was covered. Two cases were treated along with cyclosporine A and had good maternal outcomes. Four cases exhibited hemophagocytic features on their biopsies.

**Table 2:** Characteristics of patients with SLE in the literature with HLH during pregnancy.

Agey	Gestationalage	Associated factors	Clinical presentation	Treatment	Mode of delivery	Maternal Fetal		Bone marrow	Reference
**28**	22	SLE	Fever, pancytopenia, elevated triglycerides, ferritin, LDH. developed preeclampsia	IVIg, IV methylprednisolone, oral prednisone	Cesarean at 30 wk for Prom	Health	Health	hemophagocytosis	Pérard et al. (32)
**28**	5	SLE	Headaches, fevers, fatigue, and arthralgias. low C3 and elevated liver enzymes, leukopenia and thrombocytopenia	hydroxychloroquine, aspirin, prednisone, azathioprine, methylprednisolone, etoposide and dexamethasone, IVIg	Spontaneous abortion	Death	Abortion	hemophagocytosis	Parrott et al. (33)
**20**	10	SLE	NA	Corticosteroids, Cyclosporin A	Spontaneous abortion	Health	Abortion	NA	Song et al. (34)
**23**	12	SLE	Fever, elevated liver enzymes, triglycerides, CD25, ferritin, triglycerides, pancytopenia,	Corticosteroids, IVIG	Medical abortion at 19 wk	Health	Abortion	hemophagocytosis	Liu et al. (35)
**22**	24	SLE/CMV	Fever, elevated liver enzymes, ferritin, triglycerides, CD25, anaemia, thrombocytopenia	Methylprednisolone and IVIG treatment failed, remission with methylprednisolone and IVIG after delivery	29, Vaginal delivery for Prom	Health	Death	Normal	Liu et al. (35)
**31**	14	SLE	Fever, elevated liver enzymes, ferritin, CD25. Pancytopenia	Remission with corticosteroids, IVIG, Cyclosporin A	Medical abortion at 18 wk	Health	Abortion	hemophagocytosis	Liu et al. (35)
**27**	36	SLE	Fever, elevated liver enzymes, ferritin, triglycerides, CD25, pancytopenia	Remission with dexamethasone, IVIG, etoposide	Cesarean at 38 wk	Health	Health	Normal	Liu et al. (35)

Prompt diagnosis is challenging due to its varying clinical characteristics and low incidence of pregnancy-related HLH. The diagnosis is still based on the HLH 2004 standard; however, it has not been widely validated (10, 36). Diagnosis requires the detection of an HLH-associated mutation or the presence of at least five of the following eight criteria: fever >38.5 °C; ferritin >500 ng/ml (levels >3,000 ng/ml are more suggestive); two peripheral blood cytopenia; hypertriglyceridemia, and/or hypofibrinogenemia; hemophagocytosis in the bone marrow, spleen, lymph node, or liver; splenomegaly; low or absent natural killer (NK) cell activity; and elevated soluble CD 25 (37). Our case met all the indicators of the criteria. Mayama et al. (38) suggested that measuring soluble IL-2 receptor levels was the most specific diagnostic criterion for HLH. However, this indicator was normal in our patient. Hyperferritinemia was the most indicative of HLH. According to previous reports, peak serum ferritin levels >10,000 ng/ml are 90% sensitive and 96% specific for diagnosing HLH (39). However, people used the ferritin-level decline as a decent prognostic indicator for HLH patients (40). HLH was found by bone marrow tests, while one study showed that approximately 30% of the biopsies of patients showed normal recently (41). In our review, seven cases were normal in bone marrow tests. One case showed hemophagocytic features in the second bone marrow puncture. Hence, it is necessary to double-check these features in highly suspected patients.

Currently, immunosuppression, immune modulation, and chemotherapy, followed by hematopoietic stem-cell transplant, are the treatment regimens for adult patients (42). The HLH-94 regimen, which includes dexamethasone, etoposide, and cyclosporine A, is standard therapy for HLH (10). Suppressing the life-threatening inflammatory response and treating the underlying cause are the aims of treatment in pregnancy. The most common treatment during pregnancy is high doses of corticosteroids, which is relatively safest as they are inactivated in the placenta. Steroids help control life-threatening hyperinflammation due to their cytotoxicity to lymphocytes and inhibition of cytokine expression (43). We found that 95% of patients in our review were treated with corticosteroids. In the seven cases secondary to SLE, three cases were successfully treated with high-dose corticosteroids alone. Two cases had good feto-maternal outcomes, while the other newborns delivered through the vagina due to premature rupture of membranes at 29 weeks of gestation died of premature birth. Therefore, corticosteroid therapy helped maintain pregnancy stability and was considered for fetal safety. In this study, it was speculated that the inflammatory storms of the patients were so severe that removing the “trigger” was insufficient. It is difficult to have good efficacy due to a combination of the genetic mutation and SLE. This may be associated with the ineffectiveness of corticosteroids/IVIG in our patients.

Etoposide has been proven to induce prolonged remission of HLH and has become a key component of treatment protocols in nonpregnancy-related conditions, especially on the basis of corticosteroid use (44). Its use is controversial in pregnant women since it is a potential teratogenic drug and induces secondary malignancies, particularly acute myeloid leukemia (45). In a 2016 study with mice, the results indicated the potential adverse effects on fetal ovarian development (46). The study by Song et al. (34) showed that six of 13 patients with pregnancy-related HLH who used etoposide all achieved remission, and the fetus had no congenital malformations. Instead, 10 patients who used methylprednisolone/IVIG were effective in only two patients. Thus, etoposide can be actively considered an alternative treatment for patients without a response to corticosteroid/IVIG therapy. Song et al. also reported that two patients associated with autoimmune diseases responded effectively to corticosteroids and cyclosporine A. Several scholars explained that if steroids do not respond immediately, parenteral administration of cyclosporine A may be considered (47). In this study, the corticosteroids/IVIG did not perform well until the termination of pregnancy and administration of cyclosporine A. However, since the two processes were almost simultaneous, further literature is needed to support the specific cause of the obvious remission in patients. The burden on the organs of pregnant women may be increased if pregnancy is in a highly inflammatory state (48). This may be the main reason why the patient experienced elevated blood pressure and cardiac function impairment on the day of labor induction.

In previous studies, the termination of pregnancy seemed to be an effective treatment method. It may prevent the deterioration of the maternal condition and allow for timely chemotherapy. Shukla et al. reported a patient started improving on the next day of spontaneous abortion (23). However, this is still under debate. Giard et al. reported that a patient who had appropriate treatment with etoposide and dexamethasone who died on day 48 after spontaneous abortion (49). Additionally, many cases with conservative treatment achieved complete remission without the termination of pregnancy. Close monitoring of maternal and fetal conditions and timely termination of pregnancy are essential. Termination of the pregnancy should also be considered if there is no response to medication during pregnancy.

HLH can behave as a transitory self-limiting disease and recover spontaneously or disappear once the primary cause has been treated, or it can immediately progress toward a fatal outcome (50). Prognosis partially depends on gestational age. Of the 66 cases, 12 cases (18%) were in the early trimester, and two of the patients died; 31 cases (47%) were in the middle trimester, and four of the patients died; one case was lost to follow-up; 22 cases (36%) were in the late trimester, and 10 patients died; and one case was not mentioned. The second and third trimesters of pregnancy were the most common onset time of HLH. Nine of 10 pregnancies in the third trimester of pregnancy were terminated by cesarean section, and the surgery may further destroy the immunity of the patient leading to the high mortality. The appropriate method for terminating pregnancy deserves further consideration.

In FHLH, hematopoietic stem cell transplantation (HSCT) replaces the defective immune system with normally functioning cells, resulting in permanent control of the disease (7, 51). HSCT is generally recommended for patients with central nervous system involvement, relapsed/refractory disease, or proven familial disease (52). Although the patient is combined with genetic mutations, the main clinical manifestations are similar to those of MAS. Close monitoring and timely follow-up are necessary. It appears that many patients who relapse do so within a year. A monthly follow-up appears advisable during the first year of once-off therapy, followed by an annual follow-up after that (52). To prevent triggering HLH once again, pregnancy should not be carried out in the short term. Unfortunately, we cannot take HSCT into consideration if the patient has a recurrence. Donor search should be performed at the time of diagnosis because transplantation timely is a factor in the morbidity and mortality of HLH (52). Once the patient can be prepared for pregnancy after rigorous evaluation, it is recommended to strengthen monitoring during pregnancy and manage the same as SLE. Close supervision is necessary.

Because FHLH is characterized by a remarkable degree of genetic heterogeneity, the risk of a sibling carrying the disease must be considered. Clinical investigations revealed an unexpectedly high incidence of secondary MDS/AML in patients with STX11 gene mutations (53). The results of the genetic test on the family of the patient should be taken into consideration to produce a prompt diagnosis and a matched donor.

## Conclusion

Genetic factors associated with pregnancy-related HLH, especially adult-onset familial HLH, are extremely rare. The initial clinical symptoms of HLH during pregnancy lack specificity. However, early detection could reduce patient mortality. Hence, double-checking in highly suspected patients with negative bone marrow biopsy is necessary. Corticosteroids are the first choice for most patients with HLH during pregnancy. Etoposide, cyclosporine A, and termination of pregnancy may then be effective for patients with no response to corticosteroid therapy. For patients in pregnancy complicated with HLH, we should pay attention to etiological screening and consider other rare complications if conventional treatment measures are not effective. A multidisciplinary consultation should also be considered.

## Data availability statement

The original contributions presented in the study are included in the article/supplementary material. Further inquiries can be directed to the corresponding authors.

## Ethics statement

The studies involving human participants were reviewed and approved by the Ethics Committee of Qilu Hospital (Qingdao) affiliated to Shandong University. The patients/participants provided their written informed consent to participate in this study. Written informed consent was obtained from the individual(s) for the publication of any potentially identifiable images or data included in this article.

## Author contributions

WR and SY gathered the clinical information and drafted the manuscript. ZL, ZP, and HL approved the final diagnosis and formulated the therapeutic strategies. PQ and HM reviewed multiple drafts of the manuscript and gave input. All authors listed have made a substantial, direct, and intellectual contribution to the work and approved it for publication.

## Acknowledgments

We thank the pathologists, technicians, clinicians, nurses, and administrative employers who have provided support for the study.

## Conflict of interest

The authors declare that the research was conducted in the absence of any commercial or financial relationships that could be construed as a potential conflict of interest.

## Publisher’s note

All claims expressed in this article are solely those of the authors and do not necessarily represent those of their affiliated organizations, or those of the publisher, the editors and the reviewers. Any product that may be evaluated in this article, or claim that may be made by its manufacturer, is not guaranteed or endorsed by the publisher.
